# Differences in cognitive performance and neuroanatomy according to Alzheimer's disease pathophysiology

**DOI:** 10.1055/s-0046-1817040

**Published:** 2026-03-11

**Authors:** Isadora Cristina Ribeiro, Brenda Costa Gonçalves, Ítalo Karmann Aventurato, Marjorie Cristina Rocha da Silva, Liara Rizzi, Ana Luiza Gonçalves Rochetti, Gustavo Bruniera Peres Fernandes, Fernando Cendes, Marcio Luiz Figueredo Balthazar

**Affiliations:** 1 Universidade Estadual de Campinas, Instituto Brasileiro de Neurociências e Neurotecnologia, Departamento de Neurologia, Campinas SP, Brazil.; 2 Department of Neuroscience, Mayo Clinic, Jacksonville, Florida, USA.; 3 Hospital Israelita Albert Einstein, São Paulo SP, Brazil.

**Keywords:** Biomarkers, Cognition, Neuroimaging

## Abstract

**Background:**

The diagnosis of predementia stages indicates an increased risk of progression to dementia. The Amyloid, Tau, and Neurodegeneration AT(N) classification considers measurements of altered proteins and the presence of neurodegeneration to classify the risk groups regarding the pathophysiology of Alzheimer's disease (AD). The cognitive and anatomical characteristics of the patients in the predementia stage according to the AT(N) classification are not fully understood.

**Objective:**

To investigate whether there are differences in the clinical and anatomical profiles among older adults in the predementia stage according to the ATN classification, and to investigate the associations involving cognition and cortical thickness and subcortical volume in the AT(N) groups.

**Methods:**

In total, 72 older adults with subjective cognitive decline and mild cognitive impairment were allocated to groups according to the AT(N) classification (AD continuum: n = 37; suspected non-AD pathophysiology: n = 8; normal biomarkers: n = 27). The participants were investigated through cognitive tests, magnetic resonance imaging scans, and cerebrospinal fluid analyses. We used multivariate and univariate analyses with post-hoc testing to verify differences among groups. In addition, linear regressions were performed to verify the interactions involving cognition and gray matter metrics.

**Results:**

The suspected non-AD pathophysiology group showed worse performance in attention/executive function than the AD continuum and normal biomarkers groups ( *p* = 0.04). However, the ATN classification groups did not differ in terms of cortical thickness ( *p* > 0.05). In addition, in the AD continuum group, memory was associated with left fusiform gyrus thickness ( *p* = 0.000 uncorrected; r = 0.238).

**Conclusion:**

Cognition, but not gray matter metrics, differs among AT(N) classification groups. Memory is associated with cortical thickness in patients with positive amyloid Beta (AD continuum).

## INTRODUCTION

Alzheimer's disease (AD) is a gradual neurodegenerative process that begins decades before the onset of clinical symptoms. 1 2 Due to the prospect of an increase in the number of dementia cases, 3 the study of predementia stages and biomarker alterations has gained increasing importance in recent years, 4 since the early interventions can alter the clinical trajectory or delay progression to dementia. 5 6 7 Subjective cognitive decline (SCD) and mild cognitive impairment (MCI) may be earlier symptomatic stages of AD: 8 9 SCD individuals present with cognitive complaints but with preserved cognitive performance, 10 while MCI includes individuals with impaired cognitive performance but preserved functional independence. 4 11 12 These predementia stages are defined by clinical aspects. 13

Recently, for research purposes, there was a proposition to define AD in biological terms, considering changes in the amyloid Beta (Aβ) peptide, phosphorylated Tau protein (pTau), and neurodegeneration. Accordingly, the Amyloid, Tau, and Neurodegeneration (AT(N)) classification was proposed to incorporate the measurements of these proteins and neurodegeneration. According to Jack et al., 13 “A” (aggregated Aβ or associated pathological state) can be defined by cerebrospinal fluid (CSF) Aβ42, the Aβ42/Aβ40 ratio, or amyloid positron-emission tomography (PET); T (aggregated Tau or associated pathological state), by CSF pTau or Tau PET; and N (neurodegeneration or neuronal injury), by structural magnetic resonance imaging (MRI), ^18^ F-fluorodeoxyglucose positron emission tomography ( ^18^ F-FDG PET), or CSF total tau (t-tau). In this classification, the combinations of these biomarkers can be grouped into three main categories: the AD continuum, when there is a change in the Aβ peptide (A + T ± N ± ); suspected non-AD pathophysiology (SNAP), when the Aβ is normal but there is a change in pTau protein or evidence of neurodegeneration (A–T + N–, A–T–N + , or A–T + N + ); and normal biomarkers (NB), when there is no detectable alteration in these proteins, nor any evidence of neurodegeneration. 13

Thus, it is possible to describe patients in terms of their clinical diagnosis (referring to the stages of AD: SCD, MCI, and dementia) and in terms of the observed pathophysiological changes (such as the presence of biomarkers and neurodegeneration: AD continuum, SNAP, or NB). Although it is known that the diagnosis of predementia stages is associated with an increased risk of progression to dementia, the cognitive and anatomical characteristics of these groups, when subdivided according to the AT(N) classification, are not yet fully understood. Different studies have examined the cognitive profiles of individuals. In individuals with preserved cognition, no differences were observed between the SNAP group and the A − N− or A + N− groups. 14 However, there were differences in logical memory, attention, processing speed, and executive function between the SNAP group and controls. 15 16 Furthermore, the A + T+ individuals showed worse memory, executive function, and processing speed compared to those who were positive or negative for a biomarker. 17 Regarding MCI, cognitive differences between A+ subjects and those with NB were observed, but not between the SNAP and the other groups in terms of memory, executive function, and language. 18 Much less is known about SCD, such as whether this potentially predementia condition has different cognitive profiles according to the AT(N) classification.

In addition, differences in brain anatomy according to the AT(N) classification remain unknown. Patients in the AD continuum could be expected to have a profile more like that of patients with AD dementia, that is, a predominance of amnesic symptoms and atrophy of mesial temporal structures. 19 It can be assumed that SNAP patients have a cognitive profile in which non-amnesic changes predominate and, potentially, atrophy of extratemporal structures. On the other hand, this may not necessarily be true. In routine medical and neuropsychological care, these individuals often present with similar clinical features (memory complaints), but they may exhibit different pathophysiological profiles. 19

The investigation of cognitive and anatomical characteristics according to the pathophysiology of AD in patients with SCD and MCI contributes to the early identification of alterations in predementia stages, enables the application of earlier and potentially more effective interventions, and corroborates the understanding of disease staging and progression, enabling a more personalized and targeted treatment approach. In addition, it can help clinicians select patients who should undergo CSF or PET biomarker testing, especially in settings where access to these techniques is restricted, such as Brazil and other Latin American countries. 20 Furthermore, it advances the understanding of this population, who has not been previously investigated in this context, as it is less studied. 21 In the current study, we investigated if patients in mild or very mild and potentially predementia stages (SCD and MCI) have different clinical and anatomical profiles, considering the pathophysiology of AD determined by CSF biomarkers. Furthermore, we investigated whether there were associations involving cognitive measures and brain cortical thickness and subcortical volume.

## METHODS

The present is a cross-sectional study approved by the Ethics in Research Ethics Committee of Universidade Estadual de Campinas (under CAAE no. 24898619.8.0000.5404). All participants signed an informed consent form explaining in detail the aims of the research, as well as the risks and analyses of the biological material. All experiments were conducted in accordance with the Declaration of Helsinki.

### Participants

Volunteers for participation in the research were recruited through an online questionnaire posted on the university's website, which served as a screening tool to select participants who met the inclusion and exclusion criteria. The recruitment period for the study was from August 2020 to August 2021. The inclusion criteria were: patients aged ≥ 60 years presenting with or without memory complaints. The exclusion criteria were the presence of other neurological or psychiatric diseases, traumatic brain injury resulting in loss of consciousness, drug or alcohol addiction, prior chronic exposure to neurotoxic substances, an AD diagnosis, score ≥ 2 on the Fazekas scale, or score < 17 points on the Montreal Cognitive Assessment (MoCA). 22

The participants who met the selection criteria were included and investigated through a cognitive assessment that consisted of a neurological consultation to assess the clinical symptoms and cognitive tests administered by a neuropsychologist. This cognitive assessment aimed to diagnose the patient within the AD continuum (SCD, MCI, or control—preserved cognition) and assess cognitive performance.

The SCD subjects were diagnosed using the modified core criteria of the SCD-initiative (SCD-I) Working Group Criteria for SCD. 10 We included individuals with memory complaints or persistent concerns of subjective cognitive decline for at least 1 year or who reported having a memory complaint that interfered with their daily activities on the Memory Complaint Scale. 23 The SCD individuals presented cognitive performance within the average range for age and education in all cognitive tests.

The MCI diagnosis was established using the core criteria of the National Institute on Aging/Alzheimer's Association (NIA/AA )for MCI. 24 We considered cognitive performance altered when the subjects obtained a Z-score < -1.5 in any cognitive test, or a Z-score between -1 and -1.5 in at least 2 cognitive tests that measured the same cognitive function, or a Z-score between -1 and -1.5 in at least 3 cognitive tests that measured different cognitive functions. 2 10 All tests are validated for the Brazilian population, and age and schooling were considered to classify performance. 25 All participants had scored < 5 on the Pfeffer's Functional Activities Questionnaire. 26

The controls were the participants who did not present complaints of cognitive or memory loss, nor significant alterations in cognitive tests.

The SCD and MCI participants underwent the second and third steps: MRI scans (to investigate brain anatomy) and CSF sample collection (to determine the AT(N) classification: A: CSF Aβ42; T: CSF pTau; and N: CSF tTau). The controls were not included in the analysis because they could not undergo CSF collection according to our ethics committee. Therefore, we could not classify them according to AT(N) classification.

**Figure 1:**
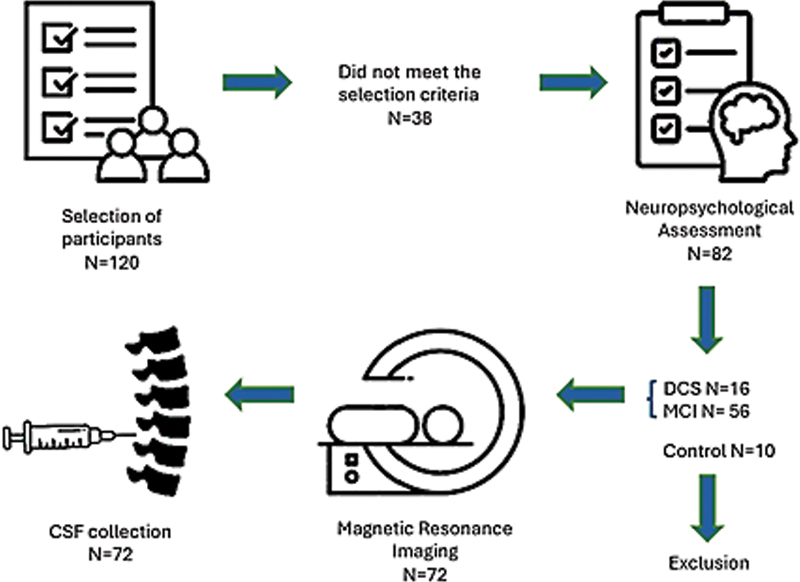
Steps of data collection of the study and number of participants included.

Figure 1 shows the steps of the study and the number of participants who completed each step. After the recruitment, the participants underwent the evaluation steps, which were scheduled in consecutive weeks following screening and enrollment: week 1–cognitive assessment; week 2–MRI; and week 3–CSF collection. All procedures, consultations, and examinations were conducted at the Clinical Research Center and the Teaching Hospital of Universidade Estadual de Campinas.

### Cognitive assessment

The main functions evaluated were global cognition, memory (verbal episodic and incidental visual), executive functions, language, and visuospatial skills. The cognitive tests used are listed below. The MoCA was used to evaluate global cognition, 22 and the Memory Complaint Scale, to screen for memory problems. 27

The Rey's Auditory Verbal Learning Test (RAVLT), which uses subitem encoding (A1 to A5, immediate recall [A6], delayed recall [A7], and recognition [excluding false positives]), was employed to evaluate verbal episodic memory. 28 The Rey-Osterrieth Complex Figure Test evaluated incidental visual (immediate and delayed) memory. 29 The Verbal Fluency (VF) Test (phonemic VF [FAS] and semantic VF [animal category]) was used to measure language and executive functions, such as working memory, self-monitoring, mental inhibition, and flexibility, as well as aspects of semantic memory, such as the ability to evoke words. 30 The Trail-Making Test, parts A and B (TMT A and B), assesses sustained attention, alternate attention, and executive function (inhibitory control and cognitive flexibility). 31

We grouped the cognitive tests into composites based on the cognitive functions: memory, attention/executive functions, and verbal fluency. The memory composite was the average of the Z-scores on the RAVLT test (total score: A1 to A5; A6; A7; and recognition) and the average of the Z-score of the immediate and delayed memory scores on the Rey Complex Figure test. The composite of attention/executive functions was determined by the average of the Z-score of the times (in seconds) of TMT A and B. Finally, the average Z-score of the phonemic VF (PVF) and semantic VF (SVF) tests formed the VF composite.

### Magnetic resonance imaging

#### *Image acquisition and processing*

The MRI scans were acquired on a Philips Achieva 3.0T Intera scanner (Philips Healthcare). High-resolution 3-dimensional T1-weighted images were obtained with isotropic 1-mm voxels in the sagittal plane (240 × 240 × 180; voxel size: 1 × 1 × 1 mm ^3^ ; TR/TE: 7/3.201 ms; and flip angle: 8°). To investigate the subcortical volumes and cortical thickness, the images were processed and analyzed using the FreeSurfer software (free and open source), version 7.3.0. 32 All images underwent visual inspection to ensure quality and to verify the accuracy of the segmentation using Freeview, the FreeSurfer's primary interactive graphical viewer used to visualize, edit, and annotate neuroimaging data. The values of the subcortical volumes were adjusted for each participant's estimated total intracranial volume (eTIV). 33 No normalization was applied to cortical thickness measures. Regions of interest were defined according to the Harvard–Oxford Atlas, and they are listed in **Supplementary Material 1** (available at https://www.arquivosdeneuropsiquiatria.org/wp-content/uploads/2025/12/ANP-2025.0089-Supplementary-Material-1.docx ).

### Cerebrospinal fluid analysis

The CSF collection from participants with clinical diagnoses of SCD and MCI was performed in the sample collection area of the university hospital. A total of 4 mL of CSF was collected via lumbar puncture performed by a trained physician. For the procedure, a 10-mL polypropylene tube was used. The sample was then aliquoted into 0.5-mL portions in 2-mL cryogenic tubes and stored at -80°C until analysis.

Biochemical analyses were conducted at the Clinical Laboratory of Hospital Israelita Albert Einstein, in the city of São Paulo. Quantification of Aβ1-42, pTau181, and tTau proteins was performed using immunoassays on the Roche cobas (Roche Diagnostics International Ltd.) analyzer. For the detection of amyloid positivity, the Elecsys β-Amyloid (1-42) CSF II kit (Roche Diagnostics International Ltd.) was used. Tau positivity was determined using the Elecsys Phospho-Tau (181P) CSF kit (Roche Diagnostics International Ltd.), and neurodegeneration, based on tTau levels, was assessed using the Elecsys Total-Tau CSF kit (Roche Diagnostics International Ltd.). The reference values used to classify the AT(N) profile were as follows: βA < 1,000 pg/mL; pTau181 > 27 pg/mL; and tTau > 300 pg/mL, following the manufacturer's instructions.

### Statistical analysis

The data were tested for normality and homoscedasticity. 34 35 All analyses were conducted controlling for sex, age, and years of schooling as covariates. A multivariate analysis of variance was performed to evaluate the differences in compounds of cognitive composites (memory, attention/executive functions, and VF) among the AT(N) groups: NB, SNAP, and AD continuum. If the multivariate tests were significant, we further performed univariate analyses. 36

We used linear regression models based on ordinary least squares (OLS) to identify differences in subcortical volumes and cortical thickness among the AT(N) groups. 37 Subsequently, an analysis of variance was performed on the fitted models to decompose the total variability of the dependent variables (subcortical volumes and cortical thickness) and to test the statistical significance of the main effects and interactions among factors. 36 To control for false positives due to multiple comparisons, we applied a false discovery rate (FDR) correction using the Benjamini–Yekutieli (TSBKY) method, with a significance threshold of α = 0.10. 38

To investigate whether the relationship involving cognitive composites (memory, attention, and executive function) and gray matter measures differs across AT(N) subtypes, we also used OLS linear regression models including an interaction term between the cognitive composite and AT(N) group. 37 Each model included as the dependent variable the thickness or volume of a specific cortical structure, and, as predictors: the cognitive composite, the AT(N) group, and the composite versus group interaction. Subsequently, an analysis of variance was performed on the fitted models, 36 and the results were corrected for multiple comparisons using the FDR (TSBKY; α = 0.10). 38 All analyses were performed in the Python (free and open source) software, version 3.9, using the Statsmodels (version 0.13.3) and Pandas (version 1.5.1) packages.

## RESULTS

Our data met the assumptions of normality and homogeneity of the sample. The data were presented in terms of mean and standard deviation values. After the screening process (N = 120), 38 volunteers were not selected because they did not meet the inclusion criteria, and 10 individuals could not participate because their control diagnosis hindered a CSF examination ( Figure 1 ). Therefore, we evaluated 72 older adults (27 men and 45 women) aged 64.13 ± 6.28 years and with 13.52 ± 4.28 years of schooling. Table 1 describes the cognitive performance stratified by predementia stages (since these values determine the clinical diagnosis). The stages did not differ regarding sex, age, and schooling ( *p* > 0.05; **Supplementary Material 2** –available at https://www.arquivosdeneuropsiquiatria.org/wp-content/uploads/2025/12/ANP-2025.0089-Supplementary-Material-2.docx ).

**Table 1:** Z-score regarding the cognitive tests among the study sample

		MCI (n = 56)		SCD (n = 16)	
		Mean	SD	Mean	SD
Memory composite tests	RAVLT A1 to A5	-0.38	± 1.13	0.85	± 0.77
	RAVLT A6	-0.64	± 1.24	0.61	± 0.81
	RAVLT A7	-0.66	± 1.26	0.67	± 0.73
	RAVLT recognition	-0.33	± 1.26	0.63	± 0.55
	RCFT immediate recall	0.22	± 1.26	1.09	± 1.39
	RCFT delayed recall	0.12	± 1.26	1.05	± 1.31
Attention/executive functions composite tests	TMT part A	-0.89	± 1.53	0.37	± 1.08
	TMT part B	-0.51	± 1.51	0.8	± 0.85
Verbal fluency composite tests	SVF	-0.35	± 1.06	-0.1	± 0.70
	PVF	-0.29	± 1.08	0.08	± 0.80

Abbreviations: MCI, mild cognitive impairment; PVF, phonemic verbal fluency; RAVLT, Rey Auditory Verbal Learning Test; RCFT, Rey-Osterrieth Complex Figure Test; SCD, subjective cognitive decline; SD, standard deviation; SVF, semantic verbal fluency; TMT, Trail-Making Test.

The AT(N) classification included 37 older adults in the AD continuum group (32 with MCI and 5 with SCD), 8 in the SNAP group (7 with MCI and 1 with SCD), and 27 in the NB group (17 with MCI and 10 with SCD). Table 2 shows the sample characteristics according to the AT(N) classification groups.

**Table 2:** Characterization of the sample according to the AT(N) classification

	AD continuum (n = 37)		SNAP (n = 8)		NB (n = 27)	
	Mean	SD	Mean	SD	Mean	SD
Age (years)	64.35	± 5.63	64.25	± 6.81	65.37	± 6.69
Schooling (years)	14.13	± 4.46	12.75	± 4.43	13.37	± 3.77
Aβ (pg/mL)	689.65	± 158.71	1699.25	± 370.50	1234.19	± 185.45
pTau181 (pg/mL)	15.64	± 7.60	27.80	± 6.73	17.59	± 3.14
tTau (pg/mL)	180.85	± 72.02	321.14	± 66.00	208.27	± 27.62

Abbreviations: Aβ, amyloid Beta (1-42); AD, Alzheimer's disease; AT(N), Amyloid, Tau, and Neurodegeneration; NB, normal biomarkers; SD, standard deviation; SNAP, suspected non-AD pathophysiology; pTau181, phosphorylated Tau 181; tTau, total Tau.

**Figure 2:**
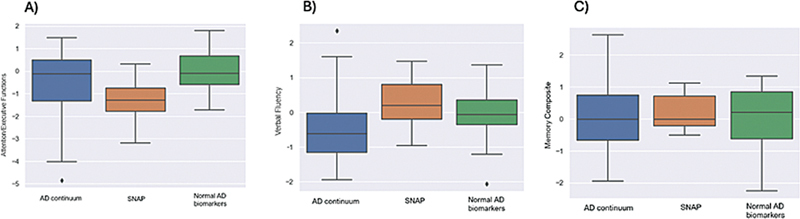
Boxplots of differences in cognitive performance according to AT(N) classification.

Regarding cognitive performance, the multivariate analysis of variance was significant for groups ( *p* = 0.02; F = 2.59). In the univariate analysis of the cognitive composites, the attention/executive functions composite differed significantly, with the boxplot showing that this difference was observed in the SNAP group ( *p* = 0.04; F = 3.23; Figure 2A ). However, no significant differences were found among the groups regarding the VF ( *p* = 0.07; F = 2.77; Figure 2B ) and memory composites ( *p* = 0.91; F = 0.08; Figure 2C ). Investigating brain regions, we found no significant differences in cortical thickness and subcortical volume among the AT(N) groups after FDR correction (R ^2^ = 0.052; adjusted R ^2^ = -0.019; F = 0.7302; *p* = 0.603; β = 2.3800; and standard error = 0.055).

In the interaction analysis regarding cognitive composites and gray matter metrics (cortical thickness and subcortical volume) across all AT(N) groups, no significant interactions were found after FDR correction (R ^2^ = 0.125; adjusted R ^2^ = 0.009; F = 1.075; *p* = 0.392; β = 2.3652; and standard error = 0.060). **Supplementary Material 3** (available at https://www.arquivosdeneuropsiquiatria.org/wp-content/uploads/2025/12/ANP-2025.0089-Supplementary-Material-3.docx ) shows all interactions identified by the analysis of variance on the adjusted models, considering *p* < 0.05, presenting both uncorrected *p* -values and those corrected for multiple comparisons. No interactions were observed for subcortical volume, only for cortical thickness.

**Figure 3:**
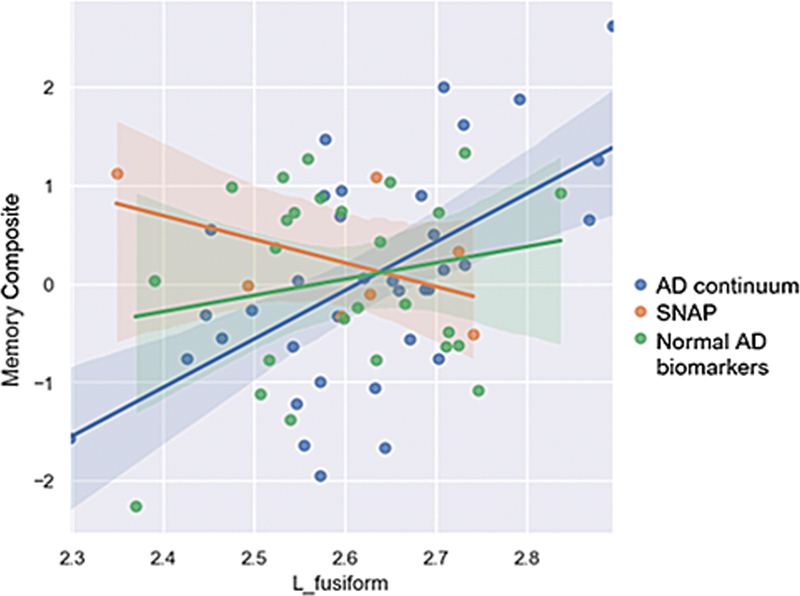
Correlations between Memory Composite and left fusiform gyrus in the three groups.

Considering uncorrected *p* -values < 0.001 as indicative of potential effects of cognitive composites on the gray matter metrics of the AT(N) groups, we observed associations between the memory composite and cortical thickness in the left fusiform gyrus ( *p* = 0.000946; F = 6.262; R ^2^ = 0.238). The interaction coefficients and group effects were estimated relative to the AD continuum group. This relationship is illustrated in a scatter plot with linear regression lines stratified by AT(N) group ( Figure 3 ).

## DISCUSSION

The current study's questions were whether there are differences in cognitive performance and neuroanatomy among potential predementia individuals in the earliest disease stages, subdivided according to AD pathophysiology. We also investigated associations between cognitive performance and gray matter thickness.

We found that the SNAP group performed worse in attention/executive tests than the AD continuum and NB groups. These results confirm our hypothesis that SNAP patients exhibited a cognitive profile in which non-amnesic changes predominate, that is, poor performance on attention/executive tests. However, we did not find significant extratemporal atrophy in this group. Our findings contrast with those of previous studies, such as the one by Ebenau et al., 39 which investigated the relationship between the AT(N) classification and the risk for dementia in patients with SCD. Their findings indicated that the SNAP group, over time, was associated with a more pronounced decline in memory tests (immediate and delayed RAVLT) than the other groups. On the other hand, the AD continuum group presented a more significant decline in memory, attention, and executive functions tests compared to the SNAP and NB groups.

In other longitudinal studies conducted by Ebenau et al. 40 and Blacker et al., 25 in a sample of SCD, a more pronounced decline in attention and executive functions was observed in those individuals who, over time, changed their amyloid status in the AT(N) biomarker profile, that is, they went from NB (A-) to AD continuum (A + ). The clinical implications of these AT(N) biomarker profiles are still poorly understood, as previous studies have shown widely variable results. Longitudinal studies 39 40 41 42 have shown that in the MCI-SNAP group, progression to dementia ranged from 0 to 56% at 2 to 3 years of follow-up. In addition, Petersen et al. 43 reported a higher conversion rate among the SNAP subjects compared to the individuals on the AD continuum. Caroli et al. 42 found a similar cognitive decline in the SNAP and AD continuum groups, and other authors 44 45 46 found a significantly smaller cognitive decline in the SNAP compared to the AD continuum group.

Another possible explanation for these divergent results is that, despite differences in biomarker concentrations in CSF, patients are clinically similar at this very early stage of the disease. They commonly seek medical attention due to “forgetfulness.” Regarding biomarker analysis, there are still certain contradictions and differences in methods and procedures, especially in the preanalytical stage, which may lead to differences in results among different laboratories. Despite the divergent results in other investigations, performance in cognitive functions (memory, attention/executive functions, VF, and others) can be an essential tool in the differential diagnosis of people with non-AD pathological alteration and those on the AD continuum or with NB.

To our knowledge, the present is the first study to compare gray matter metrics across AT(N) classification groups, and we did not find significant differences among them, which corroborates the findings of Ekman et al., 19 showing that individuals with MCI did not differ in terms of atrophy according to AT(N) classification. In addition, we did not find significant associations regarding cognition and gray matter metrics across the AT(N) groups after correction for multiple comparisons. Nonetheless, we observed a significant ( *p* < 0.001) association between the memory composite and cortical thickness in the left fusiform gyrus within the AD continuum group.

The fusiform gyrus is commonly associated with the recognition of facial features, including memory encoding, especially on the right side. 47 In the left hemisphere, it is involved in the visual, phonological, and orthographic processing of words. 48 49 50 However, even the left fusiform may be activated during memory encoding and predict subsequent free recall of information. Dickerson et al. 51 discussed that the fusiform gyrus is involved in generating a mental image and recapitulating visual-semantic properties of a stimulus, which can be necessary for memory retrieval. Considering AD pathophysiology, Montembeault et al., 52 in a sample of patients with posterior cortical atrophy with a typical AD profile, showed a specific hypometabolism pattern in the left fusiform gyrus and inferior temporal gyrus. In this sense, the relationship between the fusiform gyrus and memory in the current study may indicate that this area may be involved in some way with the measurements evaluated by the RAVLT and Rey's Complex Figure tests in the individuals in our sample.

As limitations to the study, we point out the small sample size, particularly regarding the SCD participants and the SNAP group. These differences in group size compared to the other groups may have influenced the results, affecting the reliability of group comparisons. In addition, we highlight the fact that we analyzed patients with SCD and MCI together, which did not enable us to investigate differences between them, and the fact that we did not compare our sample with individuals without cognitive impairment, as we were not authorized to collect CSF from control participants. Using PET or the Single Molecule Array (Simoa) method to investigate biomarkers could enable the inclusion of a control group. We also include as a limitation a selection bias, since participants were recruited only from the region where the university is located; therefore, the sample does not represent populations from other locations.

Although the present study involves a small sample of the Brazilian population, it contributes to the expansion of AD research in ethnically-diverse contexts, which is particularly needed in Latin America. 21 Racial and ethnic backgrounds can influence biomarker levels, 53 cognitive performance, 54 and neuroimaging outcomes. 55 Most studies on neuroimaging, cognitive performance, and AT(N) biomarkers have predominantly focused on White populations from high-income countries, limiting the generalizability of the findings. 53 54 55 Including diverse samples is essential to identify variations related to genetic, socioeconomic, and environmental factors, and to promote greater equity in diagnosis and treatment.

In conclusion, the SNAP group showed worse performance in attention/executive function than the AD continuum and NB groups. However, the AT(N) classification groups did not differ in terms of gray matter metrics. Thus, cognition, but not gray matter metrics, differs among AT(N) classification groups. In addition, in the AD continuum group, the memory composite was correlated with left fusiform gyrus thickness, showing memory is associated with cortical thickness in patients with positive Aβ (AD continuum).
